# How Co-Stimulatory/Inhibitory Molecules Vary Across Immune Cell Subtypes in the Severity of Systemic Lupus Erythematosus Compared to Controls

**DOI:** 10.3390/biomedicines12112444

**Published:** 2024-10-24

**Authors:** Kuang-Hui Yu, Wei-Tzu Lin, Ding-Ping Chen

**Affiliations:** 1 Division of Rheumatology, Allergy, and Immunology, Chang Gung University and Linkou Chang Gung Memorial Hospital, Taoyuan 333, Taiwan; gout@adm.cgmh.org.tw; 2 Department of Laboratory Medicine, Linkou Chang Gung Memorial Hospital, Taoyuan 333, Taiwan; berry0908@cgmh.org.tw; 3 Medical Biotechnology and Laboratory Science, Chang Gung University, Taoyuan 333, Taiwan

**Keywords:** systemic lupus erythematosus (SLE), immune checkpoint molecules, co-stimulatory molecules, co-inhibitory molecules, surface expression

## Abstract

Background: Co-stimulatory and co-inhibitory molecules are critical to T cell responses and involved in the pathogenesis of systemic lupus erythematosus (SLE). This study aimed to comprehensively analyze the surface expression of these molecules in various phenotypic immune cells, comparing the differences between various levels of the severity in SLE and control groups. Methods: Peripheral blood mononuclear cells (PBMCs) were isolated using Ficoll-Paque from blood samples of severe SLE patients (treatment with immunosuppressants), mild SLE patients (excluding those with persistent proteinuria or thrombocytopenia), and healthy controls (*n* = 10 each). PBMCs were stimulated for 48 h. The cells were stained with anti-CD3, CD4, CD28, PD-1, and CTLA-4 antibodies and analyzed by flow cytometry. Differences between groups were assessed using the Kruskal–Wallis test and Mann–Whitney U-test, with median values and statistical significance (*p* < 0.05) reported. Results: The results showed that CD28 expression was significantly higher in SLE patients compared to controls, with the highest levels in mild SLE. However, CD3^+^ CD28^+^ and CD4^+^ CD28^+^ cells were more prevalent in controls (*p* = 0.032 and 0.017, respectively). Mild SLE patients exhibited the highest CTLA-4 expression, with significant differences from severe SLE and controls (*p* = 0.030 and 0.037, respectively). PD-1 expression was lowest in severe SLE but highest in mild SLE within CD3^+^ CD4^+^ cells (*p* = 0.001). After 48 h of activation, CD4^+^ CTLA4^+^ and CD3^+^ CTLA4^+^ expression levels were significantly higher in controls compared to SLE groups. Conclusions: Our study highlighted that the expression of CD28, CTLA-4, and PD-1 in lymphocytes and specific T cell subsets was various according the severity of SLE in patients, underscoring their roles in disease pathogenesis.

## 1. Introduction

SLE is a complex autoimmune disease caused by a combination of genetic, epigenetic, and environmental factors. The pathogenesis of SLE remains unclear. It is known that defects in immune tolerance, hyperactive T cells, and dysregulated B cell responses contribute to the production of pathogenic autoantibodies, all of which may impact the development of SLE [1]. Among these responses, T cell activation plays a crucial initiating role in adaptive immunity by receiving TCR signals and co-stimulatory signals [2].

Although co-stimulatory and co-inhibitory molecules cannot independently activate T cells, they are crucial for the response of naïve T cells. Their nature depends on the integration of these co-stimulatory or co-inhibitory signals. Therefore, abnormal expression levels of these immune regulatory factors inevitably affect the degree of T cell activation. T cells play an indispensable role in assisting B cells, secreting pro-inflammatory cytokines, infiltrating target tissues, and amplifying inflammation, thereby contributing to the onset and development of SLE [3].

CD28 is a co-stimulatory molecule continuously expressed on the surface of T cells. When it binds to its ligand (CD80/CD86), it generates stimulatory signals that enhance various aspects of T cell-mediated immunity. Programmed death-1 (PD-1) and cytotoxic T-lymphocyte-associated protein 4 (CTLA-4) are co-inhibitory molecules that are mostly induced and expressed on activated T cells following T cell activation. CTLA-4 inhibits sustained T cell activation and proliferation by competing with CD28 for ligands, directly antagonizing CD28, or recruiting inhibitory effectors. PD-1’s immunosuppressive function is similar to that of CTLA-4. It mainly suppresses immune responses through inhibitory intracellular signaling in effector T cells and Treg cells [4,5,6]. Studies have shown that abnormal expression of co-stimulatory and co-inhibitory molecules is associated with autoimmune diseases [7].

In our previous study [8], we posited that autoimmune diseases are driven by the adaptive immune system, with T cell activation and differentiation into CD4+ T cells representing upstream pathways in this system. Therefore, we have focused on analyzing the correlation between SNPs located on the surface of T cells—specifically CTLA4, CD28, and PD1—and SLE. Since these SNPs are situated in promoters, they could potentially influence the post-translational expression of proteins. To assess the impact of protein expression on SLE, we directly measured the expression of these surface proteins, especially on CD4^+^ T cells. This approach allowed us to more accurately evaluate the relationship between protein expression and SLE pathogenesis. Although there are already studies on the soluble or mRNA levels of CD28, CTLA-4, and PD-1 and their correlation with SLE [9,10,11,12,13], the pathogenesis of SLE remains unclear. Therefore, this study aimed to investigate the expression levels and percentages of CTLA-4, CD28, and PD-1 expressed on different phenotypic lymphocytes by flow cytometry and analyze their differences among severe SLE, mild SLE, and control groups.

## 2. Materials and Methods

### 2.1. Subjects

Severe SLE patients were defined as those treated with Azathioprine (AZA), Mycophenolate Mofetil (MMF), or Cyclophosphamide (CYC). The treatment plan for SLE depends on the severity of the disease and the organ systems involved. Antimalarials, nonsteroidal anti-inflammatory drugs (NSAIDs), and low-dose corticosteroids are used to manage mild to moderate cases. When symptoms are not adequately controlled, higher doses of corticosteroids and immunosuppressants are often required [14,15]. Patients with SLE who are treated with these immunosuppressants typically present with clinical features including renal involvement [16], hematological and neuropsychiatric symptoms [17], elevated erythrocyte sedimentation rate (ESR), and positive anti-dsDNA [17]. A higher Systemic Lupus Erythematosus Disease Activity Index (SLEDAI) score indicates a more severe disease, necessitating aggressive treatment [15]. Mild SLE patients excluded those with persistent proteinuria greater than 100 mg/m^2^/day or platelet counts consistently below 100,000/µL of blood. The control group consisted of healthy individuals not using immunosuppressants and not suffering from autoimmune-related diseases. The expression of CD28 was directly investigated in 7 severe SLE patients, 7 mild SLE patients, and 10 controls. Additionally, T cell activation experiments were conducted to investigate the expression of CTLA-4 and PD-1 in another set of 10 severe SLE patients, 10 mild SLE patients, and the same 10 control individuals.

### 2.2. Ethical Approval

The Institutional Review Board of Chang Gung Memorial Hospital reviewed and approved this study. The approval IDs were 202002097B0 and 202102018B0. All study subjects provided informed consent and the study was performed according to relevant guidelines and regulations.

### 2.3. Isolate Peripheral Blood Mononuclear Cells

Peripheral blood samples were collected and diluted with phosphate-buffered saline (PBS) to a total volume of 4 mL. The diluted samples were carefully layered onto 3 mL Ficoll-Paque PLUS (Cytiva, Marlborough, MA, USA) in a centrifuge tube and centrifuged at 2000 rpm for 15 min to separate PBMCs by density gradient centrifugation. The PBMCs were then washed twice with PBS, centrifuged at 1800 rpm for 5 min each time, and the supernatant was discarded. The cells were resuspended in 1 mL of fetal bovine serum (FBS) with 10% dimethyl sulfoxide (DMSO) and stored at −80 °C for 24 h before being transferred to liquid nitrogen for long-term storage.

### 2.4. Cell Culture

To detect CTLA-4 and PD-1, PBMCs need to be activated first. The samples were thawed in a 37 °C water bath, and 5 × 10^5^ cells were cultured in a 96-well plate by adding 200 μL of RPMI 1640 medium complemented with 10% FCS, 10% FBS, 1% Penicillin–Streptomycin solution, and 2 mM L-glutamine. Phytohaemagglutinin (PHA)-L (2 μg/mL), IL-7 (1 μg/mL), and IL-2 (50 IU/mL) were added to stimulate the cells, and the plate was incubated at 37 °C with 5% CO_2_ for 48 h. After 48 h, all cells were collected [18,19].

### 2.5. Analyze by Flow Cytometry

Place 5 × 10^5^–1 × 10^6^ un-stimulated cells and all stimulated cells into flow cytometry tubes. Add 20 μL anti-CD3_FITC (IM1281U, Beckman, Coulter, Brea, CA, USA), 10 μL anti-CD4_APC-Alexa Fluor 750 (A94685, Beckman), 10 μL anti-CD28_PC7 (B23313, Beckman), 10 μL anti-CD279 (PD-1)_APC (B30633, Beckman), and 20 μL anti-CD152_PE (IM2282, Beckman). Vortex for 3–5 s, then incubate in the dark at room temperature for 15–30 min for staining. Add 3 mL PBS, vortex for 3–5 s, and centrifuge to remove unbound antibodies. Resuspend cells in 300 μL PBS for final analysis using the flow cytometer (Navios EX flow cytometry, Beckman). Before analyzing, the fluorescence compensation was performed by using single-stained samples for calibration. Analyze 10,000 cells per sample. Expression levels are reported as MFI (median fluorescence intensity). Histograms are used to visualize the expression of individual proteins, while dot plots are used to assess co-expression with CD3+ or CD4+ cells. Additionally, percentages of CD3+ or CD4+ cells positive for each co-stimulatory/co-inhibitory molecule within the population are indicated. This method is adapted from previously established protocols for flow cytometry analysis [20].

### 2.6. Statistics

Flow data collection was based on 50,000 cells or 10,000 lymphocytes, with samples containing fewer than 300 target cells excluded from analysis. First, we confirmed whether the data groups (severe SLE, mild SLE, or control group) followed a normal distribution using the Kolmogorov–Smirnov test. Then, for data that fit a normal distribution, we removed data points that were more than 2 standard deviations away from the mean for each group to avoid the influence of extreme outliers. In contrast, for non-normally distributed data, we applied the interquartile range (IQR) method. Specifically, we excluded any values that fell outside the range of Q1 − 1.5 × (Q3 − Q1) to Q3 + 1.5 × (Q3 − Q1). Subsequently, when the data groups did not follow a normal distribution, the Mann–Whitney U-test was employed. When the data groups followed a normal distribution, an independent samples t-test was used for pairwise comparisons between groups. All these analyses were performed using SPSS 17.0 software. Subjects with low numbers of CD3^+^ CD4^+^ cells were excluded from the analysis. Graphs were generated using Excel (Microsoft office 2016). Statistical significance was determined based on the results of the Mann–Whitney U-test. Since comparisons were made among three groups (severe vs. control, mild vs. control, and severe vs. mild), statistical significance was set at *p* < 0.05/3 = 0.0167 for all analyses conducted in this study.

## 3. Results

**Figure 1:**
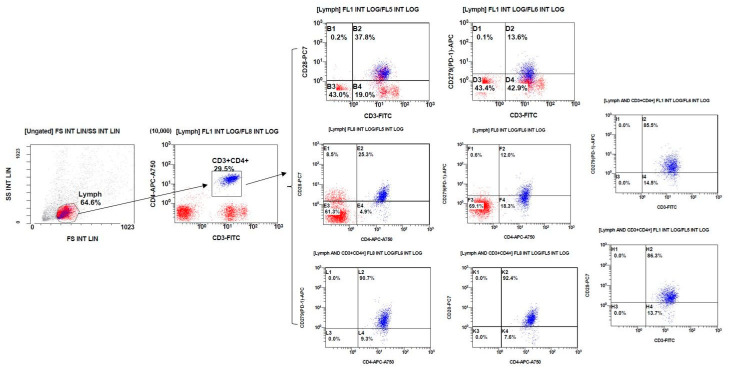
Flow cytometry analysis of lymphocytes and CD3^+^ CD4^+^ cells. Red dots: Unstained or lower-expressing cells. Blue dots: Higher-expressing cells for the relevant markers.

**Figure 2:**
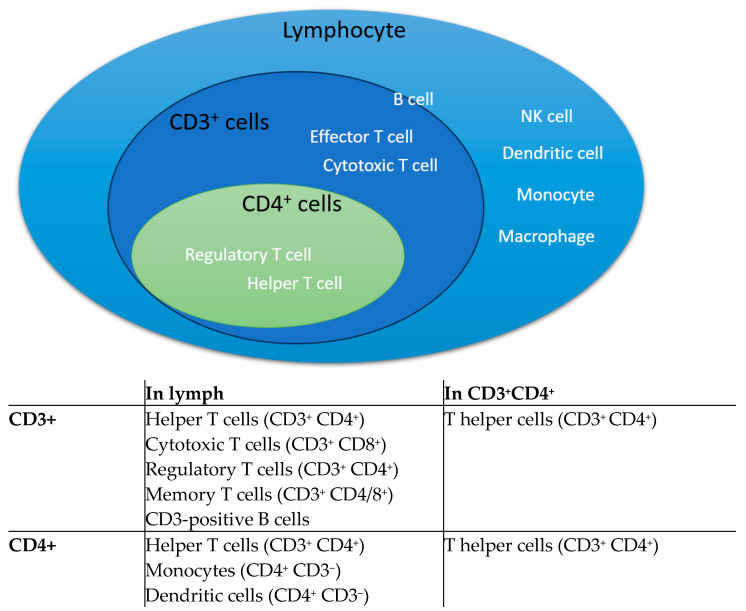
A schematic diagram of the distribution of CD3^+^ and CD4^+^ T cells in lymphocyte subsets.

First, gate the lymphocytes. Next, gate the CD3^+^ CD4^+^ cells within the lymphocytes. Then, analyze the percentage of specific phenotype cells and the protein expression levels of co-stimulatory/inhibitory molecules on these cells. The schematic diagram is shown in Figure 1. The kinds of CD3^+^ and CD4^+^ T cells that are in lymphocyte subsets are shown in Figure 2.

### 3.1. CD28 Expression Was Higher in SLE, but When Co-Expressed with CD3^+^ or CD4^+^, Its Expression Was Higher in Controls

**Figure 3:**
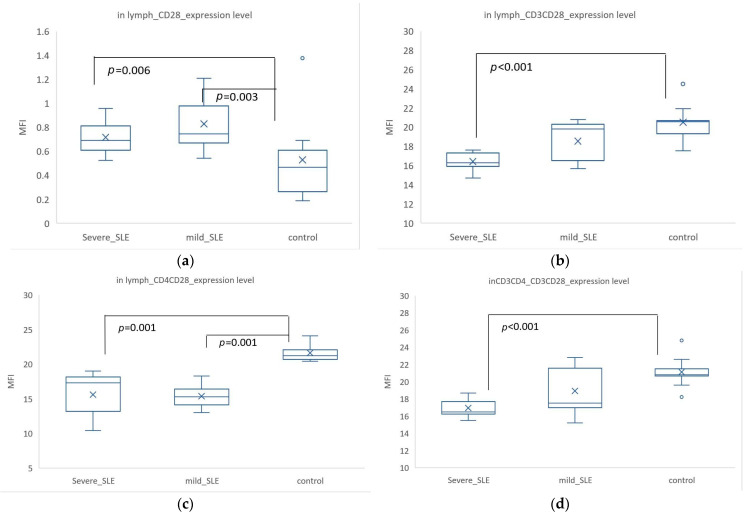
Expression levels of CD28 in different lymphocyte populations and analysis of differences among severe SLE, mild SLE, and control. Only statistically significant areas are indicated, and the *p*-values are based on the results of the Mann–Whitney U-test analysis. The ‘X’ in the box represents the mean, and the circle symbol indicate outliers. (**a**) The expression level of CD28 in lymphocytes. (**b**) The co-expression level of CD3^+^ CD28^+^ in lymphocytes. (**c**) The co-expression level of CD4^+^ CD28^+^ in lymphocytes. (**d**) The co-expression level of CD3^+^ CD28^+^ in CD3^+^ CD4^+^ cells.

The expression of CD28 in all lymphocytes showed significant differences among the three groups (inLymph_CD28_expression, *p* = 0.007, not shown in Figure). CD28 expression was higher in SLE, with mild SLE having an MFI of 0.83 ± 0.24, severe SLE having a mean rank of 0.72 ± 0.16, and control having an MFI of 0.53 ± 0.36. There were significant differences between control and mild SLE (*p* = 0.003) and between control and severe SLE (*p* = 0.006) (Figure 3a). The expression of CD3^+^ CD28^+^ cells in all lymphocytes showed a significant difference between control (MFI = 20.52 ± 1.94) and severe SLE (MFI= 16.43 ± 1.06) (*p* < 0.001) (Figure 3b). The expression of CD4^+^ CD28^+^ cells in all lymphocytes was significantly higher in control (MFI = 21.63 ± 1.25) compared to mild SLE (MFI = 15.39 ± 1.88, *p* = 0.001) and severe SLE (MFI = 15.63 ± 3.39, *p* = 0.001) (Figure 3c). In CD3^+^ CD4^+^ cells, the co-expression of CD3^+^ CD28^+^ showed a significant difference between control (MFI = 21.14 ± 1.83) and severe SLE (MFI = 16.94 ± 1.13) (*p* < 0.001) (Figure 3d). The expression of CD28 in CD3^+^ CD4^+^ cells was highest in severe SLE (MFI = 3.83 ± 1.12), with significant differences compared to control (MFI = 3.11 ± 0.37, *p* = 0.039) and mild SLE (MFI = 2.56 ± 0.58, *p* = 0.048).

Overall, the expression of CD28 in all lymphocytes and CD3^+^ CD4^+^ cells was higher in SLE compared to controls. However, when co-expressed with CD3, the percentage and expression of CD3^+^ CD28^+^ cells were higher in controls than in SLE. An interesting phenomenon was observed: the expression of CD28 in CD3^+^ CD4^+^ cells was lowest in mild SLE, with significant differences compared to control and severe SLE.

### 3.2. Mild SLE Showed Significantly Higher Expression of CTLA-4. In CD3^+^ CD4^+^ Cells, Co-Expression of CD3^+^ CTLA4^+^ Was Significantly Higher in the Control Group Compared to SLE

**Figure 4:**
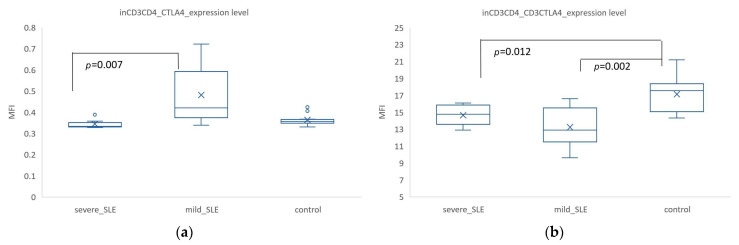
Expression levels of CTLA-4 in different lymphocyte populations and analysis of differences among severe SLE, mild SLE, and control. Only statistically significant areas are indicated, and the *p*-values are based on the results of the Mann–Whitney U-test analysis. The ‘X’ in the box represents the mean, and the circles indicate outliers. (**a**) The expression level of CTLD-4 in CD3^+^ CD4^+^ cells. (**b**) The co-expression level of CD3^+^ CTLA4^+^ in CD3^+^ CD4^+^ cells.

At the resting state, in CD3^+^ CD4^+^ cells, the overall expression level of CTLA-4 showed significant differences among the three groups, with mild SLE having the highest CTLA-4 expression (MFI = 0.48 ± 0.17), significantly different from severe SLE (MFI = 0.34 ± 0.02, *p* = 0.007) (Figure 4a). Additionally, the co-expression level of CD3^+^ CTLA4^+^ was highest in the control group (MFI = 17.11 ± 2.22), significantly higher than in mild SLE (MFI = 13.22 ± 2.53, *p* = 0.002) and severe SLE (MFI= 14.37 ± 1.45, *p* = 0.012) (Figure 4b).

The CTLA-4 expression was highest in mild SLE and lowest in severe SLE, both in all lymphocytes and in CD3^+^ CD4^+^ cells. This phenomenon was also observed in CD3+ CD4+ cells. Interestingly, the expression level of CD3+ CTLA4 in CD3^+^ CD4^+^ cells was lowest in mild SLE.

### 3.3. The Expression of PD-1 Was Significantly Lower in SLE. However, in CD3^+^ CD4^+^ Cells, PD-1 Expression Was Highest in Mild SLE

**Figure 5:**
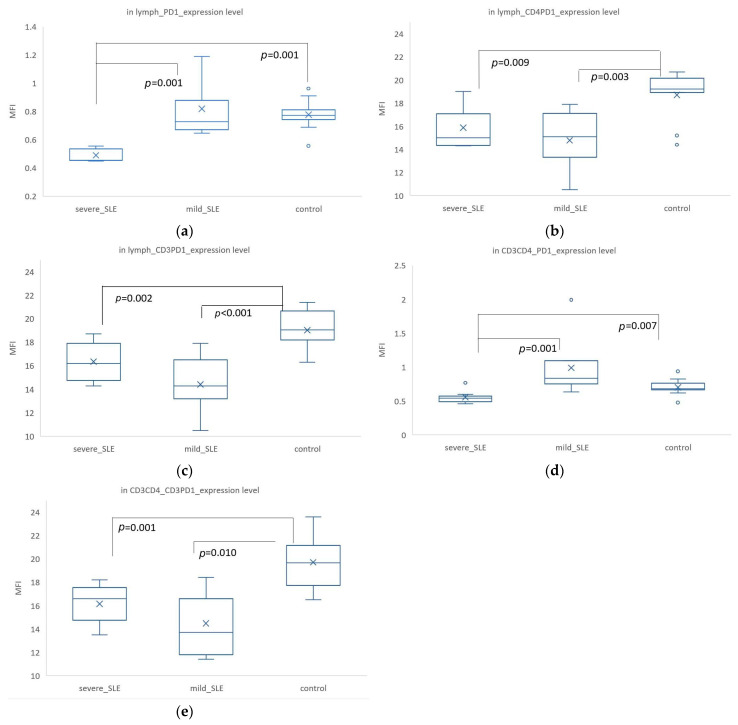
Expression levels of PD-1 in different lymphocyte populations and analysis of differences among severe SLE, mild SLE, and control. Only statistically significant areas are indicated, and the *p*-values are based on the results of the Mann–Whitney U-test analysis. The ‘X’ in the box represents the mean, and the circles indicate outliers. (**a**) The expression level of PD-1 in lymphocytes. (**b**) The co-expression level of CD4^+^ PD1^+^ in lymphocytes. (**c**) The co-expression level of CD3^+^ PD1^+^ in lymphocytes. (**d**) The expression level of PD-1 in CD3^+^ CD4^+^ cells. (**e**) The co-expression level of CD3^+^ PD1^+^ in CD3^+^ CD4^+^ cells.

The overall expression of PD-1 in all lymphocytes differed significantly among the three groups, with the lowest expression in severe SLE (MFI = 0.49 ± 0.05) and significant differences between severe SLE and control (MFI = 0.78 ± 0.11, *p* = 0.001) as well as between severe SLE and mild SLE (MFI = 0.82 ± 0.21, *p* = 0.001) (Figure 5a). The co-expression of CD4^+^ PD1^+^ was also significantly higher in the control group (MFI = 18.71 ± 2.17) compared to severe SLE (MFI = 15.66 ± 1.90, *p* = 0.009) and mild SLE (MFI = 14.78 ± 2.50, *p* = 0.003) (Figure 5b). Additionally, the co-expression of CD3^+^ PD1^+^ was significantly higher in the control group (MFI = 19.02 ± 1.85) compared to severe SLE (MFI = 16.36 ± 1.83, *p* = 0.002) and mild SLE (MFI = 14.42 ± 2.43, *p* < 0.001) (Figure 5c). In CD3^+^ CD4^+^ cells, PD-1 expression showed significant differences, with the highest expression in mild SLE (MFI = 0.99 ± 0.41), and significant differences between mild SLE and severe SLE (MFI = 0.56 ± 0.10, *p* = 0.001) as well as between severe SLE and control (*p* = 0.007) (Figure 5d). The co-expression of CD3^+^ PD1^+^ in CD3^+^ CD4^+^ cells was significantly higher in the control group (MFI = 19.70 ± 2.46) compared to mild SLE (MFI = 14.48 ± 2.67, *p* = 0.010) and severe SLE (MFI = 16.07 ± 1.74, *p* = 0.001) (Figure 5e).

The PD-1 expression was significantly lowest in severe SLE in all lymphocytes. Interestingly, in CD3^+^ CD4^+^ cells, mild SLE had the highest PD-1 expression.

### 3.4. Induced CTLA-4 and PD-1 Expression Was Significantly Increased in T Cells from Patients Compared with SLE

Compared to the data at the resting state, 48 h after activation, the expression of CD3^+^ CTLA4^+^, CD3^+^ PD1^+^, and CD4^+^ CTLA4^+^ in lymphocytes increased in the control group; in CD3^+^ CD4^+^ cells, the expression of CD3^+^ CTLA4^+^ and CD3^+^ PD1^+^ also increased. Therefore, at 48 h, the differences between SLE and healthy controls were observed in these variables.

**Figure 6:**
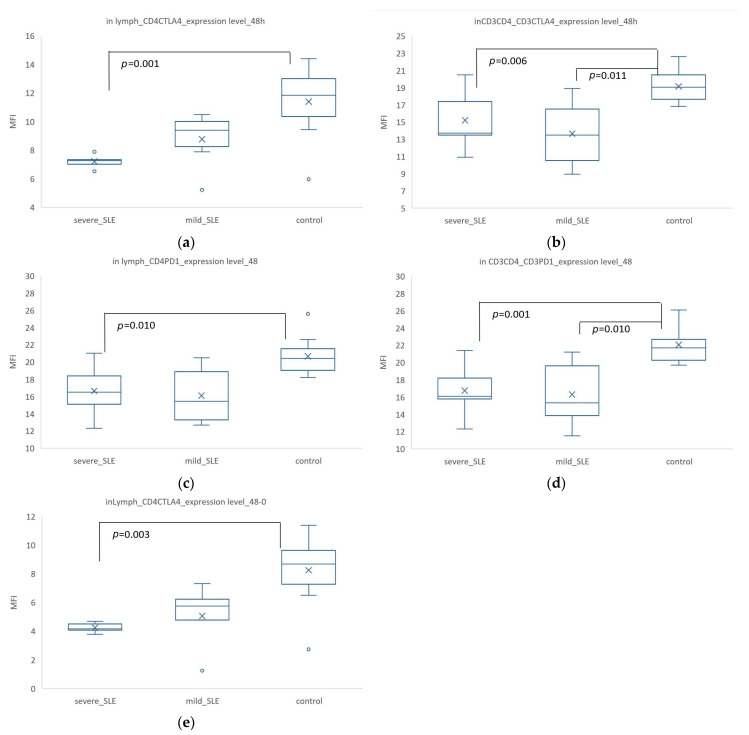
Expression levels and differential analysis of CTLA-4 and PD-1 in various lymphocyte populations after 48 h of activation. Only statistically significant differences are indicated. The *p*-values are based on the results of the Mann–Whitney U-test. The ‘X’ in the box represents the mean, and the circles indicate outliers. (**a**) The co-expression level after 48 h of CD4+ CTLA4+ in lymphocytes. (**b**) The co-expression level after 48 h of CD3+ CTLA4+ in CD3^+^ CD4^+^ cells. (**c**) The co-expression level after 48 h of CD4^+^ PD1^+^ in lymphocytes. (**d**) The co-expression level after 48 h of CD3^+^ PD1^+^ in CD3^+^ CD4^+^ cells. (**e**) The increased expression (48-0 h) of CD4^+^ CTLA4^+^ in lymphocytes.

After activation of PBMCs with IL-2, IL-7, and PHA for 48 h, the expression of CD4^+^ CTLA4^+^ in all lymphocytes showed significant differences between the control group (MFI = 11.39 ± 2.44) and severe SLE (MFI = 7.21 ± 0.50, *p* = 0.001) (Figure 6a). In CD3^+^ CD4^+^ cells, the expression of CD3^+^ CTLA4^+^ also showed significant differences between the control (MFI = 19.16 ± 1.91) and mild SLE (MFI = 13.65 ± 3.95, *p* = 0.011), as well as between control and severe SLE (MFI = 15.2 ± 3.76, *p* = 0.006) (Figure 6b). For PD-1 expression, the co-expression of CD4^+^ PD1^+^ in lymphocytes had a significance between control (MFI = 144.56 ± 1.52) and severe SLE (MFI= 10.17 ± 1.66, *p* = 0.010) (Figure 6c). The co-expression of CD3^+^ PD1^+^ in CD3^+^ CD4^+^ cells also showed significant differences among the three groups, with the highest expression in the control group (MFI = 22.07 ± 2.27), and significant differences between the control and mild SLE (MFI = 16.30 ± 3.92, *p* = 0.010) as well as severe SLE (MFI = 16.76 ± 3.35, *p* = 0.001) (Figure 6d). The increased expression of CD4^+^ CTLA4^+^ showed significant differences between the control and severe SLE (*p* = 0.003) (Figure 6e).

The detailed analysis data are shown in Supplementary Table S1.

## 4. Discussion

Since autoimmune diseases are related to the adaptive immune system and T cell activation is the cornerstone of adaptive immunity, we began to investigate gene targets associated with T cell activation, focusing on promoter and 3′UTR SNP variations. However, the biological function of genes and mRNA levels are not reliable indicators of the corresponding protein abundance [21]. Therefore, we directly used flow cytometry to detect the expression of co-stimulatory molecules on the cell surface and analyzed the differences in the expression of co-stimulatory/inhibitory molecules in PBMCs between SLE patients and healthy individuals.

In PBMCs, besides lymphocytes (T cells, B cells, and NK cells) and monocytes, there are also granulocytes (including neutrophils, basophils, and eosinophils) and a few red blood cells. Among lymphocytes, besides T cells, B cells can also express CD3 [22], so CD3^+^ cells in lymphocytes are not just T cells. Additionally, CD4^+^ T cells play an important role in the onset and progression of many autoimmune diseases [23], so we also examined the data in CD3^+^ CD4^+^ cells. We discussed the possible reasons for the differential expression of CD28, CTLA-4, and PD-1 in various immune cell phenotypes among severe SLE, mild SLE, and control based on the results.

We found that the expression of CD28 was higher in SLE, but when CD28 was co-expressed with CD3^+^ or CD4^+^, it was higher in the control group. This could be due to abnormal subset distribution and CD28 deficiency caused by the disease. The overall expression of CD28 in SLE patients was higher than in healthy controls. This may be due to abnormal lymphocyte subset distribution in SLE patients, leading to an increase or decrease in specific subsets. Non-CD3^+^ cells, such as NK cells (CD3^-^ CD56^+^), also express CD28 [24]. Research has shown that the activation-related receptor NKG2D was significantly elevated in NK cells of SLE patients [25]. Therefore, the higher overall CD28 expression in all lymphocytes of SLE patients could be due to these non-CD3^+^ cells expressing higher levels of CD28. Acknowledging the heterogeneity in SLE, it is plausible that these expression patterns of CD28 among different patient subtypes are influenced by the disease’s variability. For instance, studies have demonstrated that SLE can be divided into molecular subtypes with distinct immunological characteristics and gene expression profiles, which could affect the distribution and activation of lymphocyte subsets [26,27]. Our initial analysis indicated a subtype-specific mechanism that may reflect these findings. For example, the NEUT-H subtype described in the literature shows a heightened expression of genes associated with neutrophil activity, which could indirectly influence CD28 expression through altered immune interactions [26]. Moreover, the classification into Type 1 and Type 2 SLE based on the presence of fibromyalgia and other symptoms suggested a complex interplay between systemic symptoms and immune responses, which might impact the overall immunological profile, including co-stimulatory molecule expression [28]. This aspect underscored the importance of considering subtype-specific variations when analyzing immune markers in SLE. When co-expressed with CD3^+^ or CD4^+^, CD28 expression is higher in controls, likely due to T cell exhaustion. T cell exhaustion is a state of T cell dysfunction that arises during chronic infections and cancer, characterized by progressive loss of effector functions and proliferative capacity [29]. The features of T cell exhaustion include progressive loss of function, upregulation of inhibitory receptors, altered transcriptional and epigenetic profiles, reduced proliferative capacity, metabolic dysfunction, decreased cytotoxicity, etc. T cells in SLE patients may undergo multiple rounds of activation and proliferation, leading to some T cells losing CD28 expression and becoming CD28 negative effector T cells, a phenomenon common in chronic inflammation and autoimmune diseases. For instance, the absolute count and percentage of CD3^+^ CD8^+^ CD28^-^ T cells are significantly increased in SLE patients [30]. Additionally, CD4+ cytotoxic T cells, which lack the surface co-stimulatory molecule CD28, can promote the death of target cells through various mechanisms [31]. These CD4^+^ cytotoxic T cells are thought to play a pathogenic role in disease development. CD4^+^ T cells lacking the co-stimulatory receptor CD28 (CD4^+^ CD28^low^ cells), considered chronically activated memory/effector CD4^+^ T cells, were expanded and produced IFN-γ in patients with moderately active SLE [32]. Gofur et al. also found that the percentage of CD4^+^ CD28^+^ cells is significantly higher in controls compared to SLE patients [33]. Moreover, the number of CD3^+^ CD8^+^ CD28^-^ cells has been observed to correlate positively with disease activity in SLE [30]. Similarly, Hu et al. found fewer cells expressing CD28 in active SLE [34]. In certain conditions, such as immune aging or disease states, CD4+ cells can lose CD28 expression and become CD4^+^ CD28^-^ [35]. Thus, due to T cell exhaustion, CD28 expression is significantly higher in controls when co-expressed with CD3^+^ or CD4^+^ compared to SLE patients. We found that with increasing severity of SLE, the expression and percentage of CD28 in CD3^+^ CD4^+^ T cells significantly decreased, especially in patients with severe SLE. These findings supported the presence of T cell dysfunction and exhaustion in SLE patients, particularly those with severe disease. This may reflect the ongoing immune system activation and inflammatory state in SLE patients, leading to abnormal T cell function and regulation. Gofur et al. also found that both helper T (Th) and cytotoxic T (Tc) cells lose CD28 protein in the SLE group compared to controls, which may contribute to immune dysfunction and chronic inflammation, leading to conditions like periodontitis [33]. To summarize, in SLE patients, overall CD28 expression was higher compared to healthy controls, likely due to abnormal lymphocyte subset distribution. However, when CD28 was co-expressed with CD3^+^ or CD4^+^, its expression was higher in controls, potentially due to T cell exhaustion, where T cells lose CD28 expression after multiple rounds of activation and proliferation. This phenomenon is common in chronic inflammation and autoimmune diseases. With increasing SLE severity, the expression and percentage of CD28 in CD3^+^ CD4^+^ T cells decrease significantly, indicating T cell dysfunction and exhaustion, especially in severe cases. This aspect highlighted the importance of considering subtype-specific variations when analyzing immune markers in SLE. Therefore, we proposed that future studies should stratify SLE patients according to these molecular and clinical subtypes to explore the specific roles of CD28 and other co-stimulatory molecules in each subtype. Such stratification could provide a more nuanced understanding of the immunopathology of SLE and help tailor more effective therapeutic strategies.

At the resting state, we found that mild SLE showed significantly higher CTLA-4 expression. However, in CD3^+^ CD4^+^ cells, the co-expression of CD3^+^ CTLA4^+^ was significantly higher in controls compared to SLE. This discrepancy may be attributed to compensatory immune mechanisms and abnormal CTLA-4 expression in specific subsets. CTLA-4 expression in all lymphocytes at the resting state was highest in mild SLE, with no significant difference between severe SLE and control groups, which aligned with the expression trends in CD3+ CD4+ cells. At the resting state, CTLA-4 is expressed on regulatory T (Treg) cells, dendritic cells (DCs), and a small number of B cells and NK cells [36,37]. We found that the overall expression of CTLA-4 in all lymphocytes showed significant differences among the three groups, with notably higher CTLA-4 expression in mild SLE. This may reflect a compensatory mechanism in mild SLE patients, where enhanced Treg cell function or increased Treg cell numbers suppress excessive immune responses [38]. Additionally, Mohamed et al. found that the surface expression of CTLA-4 on peripheral blood T-lymphocytes in children with SLE was upregulated regardless of lupus activity [39]. However, our study found that this upregulation did not occur in severe SLE, which may indicate that the compensatory mechanism fails in severe SLE patients, leading to more severe disease manifestations. This finding highlighted the critical regulatory role of CTLA-4 in SLE, providing important references for future research and clinical applications. Moreover, the expression of CTLA-4 in DCs, B cells, and NK cells may also contribute to the overall expression levels, but the specific changes in SLE patients require further investigation. In CD3^+^ CD4^+^ cells, the co-expression of CD3^+^ CTLA4^+^ was significantly lower in mild SLE compared to both severe SLE patients and controls. This result indicates that, despite the higher overall expression of CTLA-4 in mild SLE patients, the expression of CTLA-4 in CD3^+^ CD4^+^ cells was relatively low. CD4^+^ T cells, also known as helper T (Th) cells, can be classified into several subsets, such as Th1, Th2, Th17, Treg, and follicular helper T cells (Tfh). This lower expression may be due to reduced CTLA-4 expression in specific subsets, as supported by Zhao et al.’s study [40], which found that the CTLA-4 levels of CD4^+^ CD25^+^ T cells and CD4^+^ CD25^+^ FoxP3^+^ Treg cells were significantly reduced in SLE patients and negatively correlated with the disease activity and severity of SLE.

Additionally, PD-1 expression was significantly lower in SLE patients compared to controls at the resting status, especially in patients with severe SLE. However, in CD3^+^ CD4^+^ cells, PD-1 expression was highest in mild SLE. This discrepancy may be related to compensatory mechanisms and changes in T cell subsets. In lymphocytes and CD3^+^ CD4^+^ T cells, PD-1 expression was significantly higher in controls compared to SLE patients. We found that PD-1 expression on the surface of CD4^+^ T cells was the lowest in severe SLE patients. Several studies have shown that in healthy individuals, PD-1 is predominantly expressed by effector memory T cells (TEM T cells) rather than terminally differentiated effector memory CD45RA^+^ T cells, and is never expressed on naïve T cells [41,42]. Additionally, studies have demonstrated that in the CD4+ T cell subsets of SLE patients, there is an increasing trend in the proportion of terminally differentiated effector memory T cells (TDEM T cells, CCR7^-^ CD45RA^+^) compared to healthy controls [43]. Thus, our results showed that among all lymphocytes, the expression of PD-1 was significantly lower in SLE patients, especially the lowest expression in severe SLE, which may be related to changes in T cell subsets in SLE patients. Specifically, in the T cell population of SLE patients, particularly those with severe SLE, a higher proportion of TDEM T cells do not express PD-1. This could partially explain why overall PD-1 expression was lower in SLE patients. Additionally, in healthy individuals, TEM T cells are the main cell type expressing PD-1. Although the proportion of TEM T cells might increase in SLE patients, these cells were more likely to be terminally differentiated (i.e., TDEM T cells), leading to reduced PD-1 expression. However, contrary to our results, a study has found that PDCD1 mRNA and soluble PD-1 were lower in controls [12]. It is important to note that the expression levels of proteins and mRNA are not necessarily the same or directly proportional. The research analyzed the correlation between the abundance of E. coli mRNAs and membrane proteins or soluble proteins and found only a moderate correlation, which did not provide significance [44]. This indicated that the relationship between mRNAs and membrane proteins or soluble proteins depends on various factors, including protein function, gene regulatory mechanisms, translation, and processing, as well as the cell’s state and environment. Future studies should further explore the functions of soluble PD-1 and membrane PD-1 and their roles in SLE. In all lymphocytes and CD3^+^ CD4^+^ cells, PD-1 expression was highest in mild SLE. The compensatory mechanism refers to the process by which the body maintains immune balance by enhancing or regulating other immune pathways in response to immune system imbalances or dysfunctions. In addition to CTLA-4, PD-1 also has a compensatory mechanism [45]. Our results showed that the expression of CTLA-4 and PD-1 was highest in mild SLE at the rest status, which could be explained by this compensatory mechanism. However, in severe SLE, the compensatory mechanism might be impaired. Additionally, there could be significant changes in immune cell subsets in severe SLE patients, such as shifts in the proportions of TEM and TDEM [43], or an abnormal increase in PD-1^+^ CXCR5^-^ CD4^+^ T cells in SLE [46]. These medications might directly or indirectly affect the expression of PD-1 and CTLA-4, resulting in their lower expression in severe SLE patients compared to mild SLE patients. Investigating these mechanisms can help better understand the pathophysiological processes of SLE and provide new insights for treatment. At the resting state, PD-1 expression in mild SLE and severe SLE was related. PD-1 is hardly expressed on naïve T cells [41,42], so the PD-1 expression observed in severe SLE could be on other immune cells. In addition to T cells, PD-1 is also expressed on the surface of NK cells, B cells, macrophages, DCs, and monocytes [47]. For example, a recent study indicated that an increase in TIM-3^+^ PD-1^+^ NK cells was positively correlated with SLE disease activity and severity [48]. However, contrary to Luo et al.’s findings [48], our results showed that PD-1 expression was significantly higher in mild SLE compared to severe SLE. Therefore, the role of PD-1 on other immune cell phenotypes and its correlation with the severity of SLE should be further investigated to fully understand the mechanisms through which PD-1 contributes to SLE pathogenesis. PD-1 exhibited a similar phenomenon to CTLA-4. At the resting state, the overall expression levels of PD-1 and CTLA-4 in all lymphocytes and CD3^+^ CD4^+^ cells were highest in mild SLE. As mentioned above, both CTLA-4 and PD-1 have compensatory mechanisms that prevent excessive immune attack and disease progression [45]. Conversely, in severe SLE patients, the low expression of PD-1 might reflect a failure in immune regulation, leading to more severe disease. This phenomenon was supported by data from 48 h post-activation, where the expression levels of CD4^+^ CTLA4^+^ and CD3^+^ CTLA4^+^ were significantly highest in the control group, and the increase in expression was significantly different from that in SLE patients. Further research could delve into the specific roles of CTLA-4 and PD-1 in different stages of SLE progression and its potential therapeutic value.

After 48 h of activation, the induced CTLA-4/PD-1 expression was significantly increased in T cells from patients compared with SLE, and the increase in CTLA-4 expression in CD3^+^ CD4^+^ cells after activation was significantly different between the control group and SLE patients. This could be due to an abnormal mechanism of CTLA-4/PD-1 expression following activation. After 48 h of activation, the expression levels of CD4^+^ CTLA4^+^ show significant differences between the control group and severe SLE patients. Although there was an increase in CD4^+^ CTLA4^+^ expression in T cells after activation in SLE patients, it was significantly lower than in the control group, and the co-expression of CD3^+^ CTLA4^+^ in SLE patients hardly increased. This indicated that despite the increased expression of CD4^+^ CTLA4^+^ after T cell activation in SLE patients, the increase was significantly less than in healthy controls. Other studies have also found that the CTLA-4 level of CD4^+^ T cells was negatively correlated with the disease activity and severity of SLE [40] and that immune rheostat dysregulation in SLE led to abnormal immune regulatory responses, resulting in reduced CTLA-4 expression in CD4^+^ T cells [49]. This suggested that the immune response in SLE was dysregulated or impaired. After 48 h, the increase in CD3^+^ CTLA4^+^ expression in CD3^+^ CD4^+^ cells in the control group was significantly higher than in SLE. CTLA-4 expression in CD3^+^ cells barely increases. This may indicate that the regulatory mechanism of CTLA-4 in SLE patients was suppressed or impaired [49], leading to excessive activation of the immune system and autoimmune responses. In SLE, PD-1 and CTLA-4 play critical roles in regulating T cell activation and maintaining immune tolerance. These co-inhibitory receptors are essential for preventing excessive immune responses that can lead to tissue damage. Although targeting CTLA-4 and PD-1 presents therapeutic potential for managing SLE, it also carries significant risks, including the possibility of immune-related adverse events. Therefore, identifying specific markers on distinct cell types is crucial. By understanding the specific roles of CTLA-4 and PD-1 in T cell regulation within the context of SLE, we can develop targeted therapies that selectively modulate immune responses without compromising overall immune defense. These findings underscored the importance of CTLA-4 in the pathophysiology of SLE and suggested it could be a potential therapeutic target.

Compared to 0 h, after 48 h, the expression of CD3^+^ CTLA4^+^, CD3^+^ PD1^+^, and CD4^+^ CTLA4^+^ in lymphocytes significantly increased in the control group. In CD3^+^ CD4^+^ cells, the expression of CD3^+^ CTLA4^+^ and CD3^+^ PD1^+^ also increased. However, in severe SLE patients, there was almost no increase. The 48 h change in lymphocyte CD4^+^ CTLA4^+^ co-expression showed significant differences among the three groups, particularly between the control and severe SLE groups, which suggested that the impaired function of CTLA-4/PD-1 expression after activation in severe SLE patients may be a contributing factor. PD-1 was moderately expressed in CD4^+^ Treg cells (CD4^+^ Foxp3^-^ T cells) and highly expressed in stimulated CD4^+^ T cells [50]. Yeo et al. also mentioned the abnormal expression of CTLA4 in SLE [49], indicating that the mechanism of CTLA-4 and PD-1 production after activation may be impaired in SLE patients. It is important to note that CD4^+^ CTLA4^+^ co-expression in lymphocytes did not solely indicate Tregs, as all mononuclear cells express CD4 protein [51], and monocytes, macrophages, and dendritic cells also express CTLA-4 protein [37], which suggested that these cells play a role in the pathogenesis of SLE and influence the severity of the disease. After 48 h of activation, the data that showed increases were almost all significantly different between the control and SLE groups, except for the expression of CD3^+^ CTLA4 in lymphocytes, which showed no significant difference. This suggested that CTLA-4 in CD3^+^ cells may not play a significant role in SLE pathogenesis or has a minor role. CTLA-4 primarily regulates immune responses in T cells, especially in CD4^+^ T cells [52]. Therefore, observing CTLA-4 expression changes in all lymphocytes may not be as significant as in specific T cell populations. This can be supported by the following results. The CD4^+^ CTLA4+ co-expression showed no significant differences at the resting state, but after 48 h of activation, there were significant differences between control, mild, and severe SLE, with control increasing the most. At the resting state, the expression levels of CD4^+^ CTLA4+ cells were similar in healthy individuals and SLE patients, indicating that CTLA-4 expression was not significantly affected by disease status under quiescent conditions. SLE is a chronic autoimmune disease characterized by persistent immune system activation and inflammatory responses. This chronic immune activation may alter the function and subtype patterns of T cells [53], making these cells unable to significantly upregulate CTLA-4 upon reactivation, unlike healthy cells. In SLE patients, T cells may be exhausted, where cells typically express high levels of inhibitory receptors such as PD-1 and CTLA-4 [54,55]. This dysfunction prevents these cells from properly upregulating CTLA-4 upon activation. CTLA-4 expression is induced after T cell activation and its protein levels are tightly regulated by various mechanisms, including ligand-induced expression, translocation to the cell surface, rapid internalization, recycling, and degradation [6]. Further investigation is needed to determine which mechanisms are abnormal and lead to disease. The dynamic expression of CD4^+^ CTLA4+ cells differed significantly between healthy controls and SLE patients, reflecting differences in immune regulatory functions and disease states. This finding aided in understanding the immunoregulatory mechanisms in SLE and may provide important insights for future therapeutic strategies.

Overall, the expression levels of CTLA-4 and PD-1 decreased in total lymphocytes but increased in specific T cell subsets after T cell activation, indicating a finely tuned dynamic regulatory mechanism of the immune system in response to and regulation of immune reactions. This phenomenon reflected the varying demands for inhibitory molecules by the immune system across different cell populations and at different stages of the immune response.

The strengths of this study lie in its comprehensive exploration of the immune phenotypes of different types of lymphocytes with SLE. However, this study has several limitations. The opposing functions of CD28 and CTLA-4 are regulated through interactions with the same ligands [4]. Future studies should analyze the ratio of CTLA-4 to CD28 expression to assess its relevance to SLE. Unfortunately, in this study, the CD28 and CTLA-4 samples came from different individuals, not allowing such an analysis. Additionally, because we used leftover samples from patients’ routine follow-ups, the number of PBMCs obtained was relatively low, and the presence of red blood cells could have slightly affected the flow cytometry detection results. Moreover, we classified the patients into severe and mild SLE without considering whether the disease was in an active state. The expression of cell surface proteins may be related to disease activity, resulting in a wider range of data. Given that protein expression can vary depending on the patient’s current health status, we did not evaluate whether the patients were in an active disease state. Future studies should consider using Spearman’s rank correlation to analyze the relationship between systemic lupus erythematosus disease activity index (SLEDAI) and protein expression levels. It is also important to consider the clinical characteristics and treatment history of patients, which may require stratified data analysis to better understand the immune features at different stages of SLE. These findings were crucial for understanding the pathological mechanisms of SLE and developing personalized treatment strategies.

## 5. Conclusions

SLE is a chronic inflammatory disease, which raises the possibility of T cell exhaustion. We cannot define the role of a protein in disease pathogenesis solely based on its well-known function. For example, CD28 is typically known to promote immune responses, yet we found that CD28 expression in CD4^+^ cells was significantly lower in SLE patients. Therefore, it was essential to explore the role of proteins in different phenotypes of immune cells to understand their involvement in disease pathogenesis. The immune response is characterized by self-regulation and negative feedback mechanisms, such as compensatory mechanisms or the increase in specific subpopulation ratios. Severe autoimmune diseases occur when compensatory regulatory mechanisms are abnormal or insufficient to counteract abnormal immune responses. The expression patterns of co-stimulatory and co-inhibitory molecules could serve as critical indicators for understanding the immunodynamic changes during the pathogenesis and progression of SLE. Future research could benefit from analyzing compensatory mechanisms and changes in specific subpopulation ratios to gain deeper insights into the regulatory mechanisms of the immune response and the characteristics of abnormal immune states. This approach could further elucidate the pathogenesis of SLE.

## Data Availability

The datasets used and/or analyzed during the current study are available from the corresponding author(s) on reasonable request.

## Supplementary Materials

The following supporting information can be downloaded at: https://www.mdpi.com/article/10.3390/biomedicines12112444/s1, Table S1: The raw data of the flow cytometry data analyzed by the Mann–Whitney U-test.

## References

[1] Bolouri N., Akhtari M., Farhadi E., Mansouri R., Faezi S.T., Jamshidi A., Mahmoudi M. (2022). Role of the innate and adaptive immune responses in the pathogenesis of systemic lupus erythematosus. Inflamm. Res..

[2] Janeway C.A., Travers P., Walport M., Shlomchik M. (2001). Immunobiology: The Immune System in Health and Disease.

[3] Suárez-Fueyo A., Bradley S.J., Tsokos G.C. (2016). T cells in Systemic Lupus Erythematosus. Curr. Opin. Immunol..

[4] Gardner D., Jeffery L.E., Sansom D.M. (2014). Understanding the CD28/CTLA-4 (CD152) pathway and its implications for costimulatory blockade. Am. J. Transplant. Off. J. Am. Soc. Transplant. Am. Soc. Transpl. Surg..

[5] Waldman A.D., Fritz J.M., Lenardo M.J. (2020). A guide to cancer immunotherapy: From T cell basic science to clinical practice. Nat. Rev. Immunol..

[6] Hossen M.M., Ma Y., Yin Z., Xia Y., Du J., Huang J.Y., Huang J.J., Zou L., Ye Z., Huang Z. (2023). Current understanding of CTLA-4: From mechanism to autoimmune diseases. Front. Immunol..

[7] He W., Wang B., Li Q., Yao Q., Jia X., Song R., Li S., Zhang J.A. (2019). Aberrant Expressions of Co-stimulatory and Co-inhibitory Molecules in Autoimmune Diseases. Front. Immunol..

[8] Chen D.P., Lin W.T., Yu K.H. (2022). Investigation of the association between the genetic polymorphisms of the co-stimulatory system and systemic lupus erythematosus. Front. Immunol..

[9] Brambila-Tapia A.J., Gámez-Nava J.I., Salazar-Páramo M., Munoz-Valle J.F., González-López L., Llamas-Covarrubias M.A., Gutiérrez-Urena S.R., Vázquez-Del Mercado M., Dávalos-Rodríguez I.P. (2011). Increased CD28 serum levels are not associated with specific clinical activity in systemic lupus erythematosus. Rheumatol. Int..

[10] Gong J.Q., Chen Z.Q., Hu Z.J., Li W.Z. (2001). Expression of CD28 mRNA in peripheral blood mononuclear cells of patients with systemic lupus erythematosus. Chin. J. Rheumat.

[11] AlFadhli S. (2013). Overexpression and secretion of the soluble CTLA-4 splice variant in various autoimmune diseases and in cases with overlapping autoimmunity. Genet. Test. Mol. Biomark..

[12] Bassiouni S., Abdeen H., Morsi H., Zaki M., Abdelsalam M., Gharbia O. (2021). Programmed death 1 (PD-1) serum level and gene expression in recent onset systemic lupus erythematosus patients. Egypt. Rheumatol..

[13] Wong C.K., Lit L.C., Tam L.S., Li E.K., Lam C.W. (2005). Aberrant production of soluble costimulatory molecules CTLA-4, CD28, CD80 and CD86 in patients with systemic lupus erythematosus. Rheumatology.

[14] Cancro M.P., D’Cruz D.P., Khamashta M.A. (2009). The role of B lymphocyte stimulator (BLyS) in systemic lupus erythematosus. J. Clin. Investig..

[15] Zagales R., Tarvin S.E., Rodriguez M. (2023). Clinical Characteristics of Pre-pubescent Patients with Systemic Lupus Erythematosus (SLE). Proc. IMPRS.

[16] López-Domínguez R., Villatoro-García J.A., Marañón C., Goldman D., Petri M., Carmona-Sáez P., Alarcón-Riquelme M., Toro-Dominguez D. (2024). Immune and molecular landscape behind non-response to Mycophenolate Mofetil and Azathioprine in lupus nephritis therapy. Res. Sq..

[17] Milone A., Parlati C., Patrone M., Riccio F., Annunziata M., Brancaccio M., Marchesano M., Fasano S., Mauro D., Ciccia F. (2024). AB1094 predictive factors of response to mycophenolate mofetil treatment in a single-center cohort of patients with systemic lupus erythematosus. Ann. Rheum. Dis..

[18] Steiner K., Waase I., Rau T., Dietrich M., Fleischer B., Bröker B.M. (1999). Enhanced expression of CTLA-4 (CD152) on CD4+ T cells in HIV infection. Clin. Exp. Immunol..

[19] Molvi Z. (2020). Activation of Human T Cells with Phytohaemagglutinin (PHA).

[20] Cossarizza A., Chang H.D., Radbruch A., Acs A., Adam D., Adam-Klages S., Agace W.W., Aghaeepour N., Akdis M., Allez M. (2019). Guidelines for the use of flow cytometry and cell sorting in immunological studies (second edition). Eur. J. Immunol..

[21] Pradet-Balade B., Boulmé F., Beug H., Müllner E.W., Garcia-Sanz J.A. (2001). Translation control: Bridging the gap between genomics and proteomics?. Trends Biochem. Sci..

[22] Nagel A., Möbs C., Raifer H., Wiendl H., Hertl M., Eming R. (2014). CD3-positive B cells: A storage-dependent phenomenon. PLoS ONE.

[23] Sun L., Su Y., Jiao A., Wang X., Zhang B. (2023). T cells in health and disease. Signal Transduct. Target. Ther..

[24] Zhuang X., Long E.O. (2019). CD28 Homolog Is a Strong Activator of Natural Killer Cells for Lysis of B7H7(+) Tumor Cells. Cancer Immunol. Res..

[25] Wang T., Wei L., Meng S., Song W., Chen Y., Li H., Zhao Q., Jiang Z., Liu D., Ren H. (2023). Coordinated Priming of NKG2D Pathway by IL-15 Enhanced Functional Properties of Cytotoxic CD4^+^CD28^−^ T Cells Expanded in Systemic Lupus Erythematosus. Inflammation.

[26] Li H., Yang P. (2022). Identification of biomarkers related to neutrophils and two molecular subtypes of systemic lupus erythematosus. BMC Med. Genom..

[27] Cui M., Li T., Yan X., Wang C., Shen Q., Ren H., Li L., Zhang R. (2021). Blood Genomics Identifies Three Subtypes of Systemic Lupus Erythematosus: “IFN-High,” “NE-High,” and “Mixed”. Mediat. Inflamm..

[28] Rogers J.L., Eudy A.M., Pisetsky D., Criscione-Schreiber L.G., Sun K., Doss J., Clowse M.E.B. (2021). Using Clinical Characteristics and Patient-Reported Outcome Measures to Categorize Systemic Lupus Erythematosus Subtypes. Arthritis Care Res..

[29] Blank C.U., Haining W.N., Held W., Hogan P.G., Kallies A., Lugli E., Lynn R.C., Philip M., Rao A., Restifo N.P. (2019). Defining ‘T cell exhaustion’. Nat. Rev. Immunol..

[30] Żabińska M., Krajewska M., Kościelska-Kasprzak K., Klinger M. (2016). CD3(+)CD8(+)CD28(-) T Lymphocytes in Patients with Lupus Nephritis. J. Immunol. Res..

[31] Takeuchi A., Saito T. (2017). CD4 CTL, a Cytotoxic Subset of CD4(+) T Cells, Their Differentiation and Function. Front. Immunol..

[32] Kosmaczewska A., Ciszak L., Stosio M., Szteblich A., Madej M., Frydecka I., Wiland P., Szmyrka M. (2020). CD4^+^CD28^null^ T cells are expanded in moderately active systemic lupus erythematosus and secrete pro-inflammatory interferon gamma, depending on the Disease Activity Index. Lupus.

[33] Gofur N.R.P., Handono K., Kalim H., Wahono C.S., Poeranto S., Barlianto W. (2020). Association of Th-Tc protein CD28+ and Periodontal Inflammation among Indonesian Women with SLE Disease. Syst. Rev. Pharm..

[34] Hu S., Tao D., He P. (2004). Expression of costimulatory molecules B7/CD28 in systemic lupus erythematosus. J. Huazhong Univ. Sci. Technol. Med. Sci..

[35] Yuan S., Zeng Y., Li J., Wang C., Li W., He Z., Ye J., Li F., Chen Y., Lin X. (2022). Phenotypical changes and clinical significance of CD4^+^/CD8^+^ T cells in SLE. Lupus Sci. Med..

[36] Kim G.-R., Choi J.-M. (2022). Current Understanding of Cytotoxic T Lymphocyte Antigen-4 (CTLA-4) Signaling in T-Cell Biology and Disease Therapy. Mol. Cells.

[37] Oyewole-Said D., Konduri V., Vazquez-Perez J., Weldon S.A., Levitt J.M., Decker W.K. (2020). Beyond T-Cells: Functional Characterization of CTLA-4 Expression in Immune and Non-Immune Cell Types. Front. Immunol..

[38] Mitsuiki N., Schwab C., Grimbacher B. (2019). What did we learn from CTLA-4 insufficiency on the human immune system?. Immunol. Rev..

[39] Mohamed E., Safaa I., Manal Y., Ibrahim I. (2007). Expression of cytotoxic T-lymphocyte-associated antigen-4 [CTLA-4; CD152] on peripheral blood T lymphocytes in systemic lupus erythematosus children. Egypt. J. Med. Microbiol..

[40] Zhao L., Zhou X., Zhou X., Wang H., Gu L., Ke Y., Zhang M., Ji X., Yang X. (2020). Low expressions of PD-L1 and CTLA-4 by induced CD4(+)CD25(+) Foxp3(+) Tregs in patients with SLE and their correlation with the disease activity. Cytokine.

[41] Petrelli A., Mijnheer G., van Konijnenburg D.P.H., Van Der Wal M.M., Giovannone B., Mocholi E., Vazirpanah N., Broen J.C., Hijnen D., Oldenburg B. (2018). PD-1^+^ CD8^+^ T cells are clonally expanding effectors in human chronic inflammation. J. Clin. Investig..

[42] Park H.J., Park J.S., Jeong Y.H., Son J., Ban Y.H., Lee B.-H., Chen L., Chang J., Chung D.H., Choi I. (2015). Correction: PD-1 upregulated on regulatory T cells during chronic virus infection enhances the suppression of CD8^+^ T cell immune response via the interaction with PD-L1 expressed on CD8^+^ T cells. J. Immunol..

[43] Piantoni S., Regola F., Zanola A., Andreoli L., Dall’Ara F., Tincani A., Airo P. (2018). Effector T-cells are expanded in systemic lupus erythematosus patients with high disease activity and damage indexes. Lupus.

[44] Masuda T., Saito N., Tomita M., Ishihama Y. (2009). Unbiased Quantitation of Escherichia coli Membrane Proteome Using Phase Transfer Surfactants. Mol. Cell. Proteom..

[45] Huang R.Y., Francois A., McGray A.R., Miliotto A., Odunsi K. (2017). Compensatory upregulation of PD-1, LAG-3, and CTLA-4 limits the efficacy of single-agent checkpoint blockade in metastatic ovarian cancer. Oncoimmunology.

[46] Lin J., Yu Y., Ma J., Ren C., Chen W. (2019). PD-1+CXCR5-CD4+T cells are correlated with the severity of systemic lupus erythematosus. Rheumatology.

[47] Ahmadzadeh M., Johnson L.A., Heemskerk B., Wunderlich J.R., Dudley M.E., White D.E., Rosenberg S.A. (2009). Tumor antigen-specific CD8 T cells infiltrating the tumor express high levels of PD-1 and are functionally impaired. Blood.

[48] Luo Q., Kong Y., Fu B., Li X., Huang Q., Huang Z., Li J. (2022). Increased TIM-3(+)PD-1(+) NK cells are associated with the disease activity and severity of systemic lupus erythematosus. Clin. Exp. Med..

[49] Yeo J., Yaung K., Law A., Wasser M., Arkachaisri T., Thumboo J., Low A.H.L., Albani S. (2023). OP0214 a multi-dimensional approach reveals a dysregulated systemic lupus erythematosus immune rheostat with an abnormal immunoregulatory response and reduced ctla4 expression in effector T cells. Ann. Rheum. Dis..

[50] Francisco L.M., Sage P.T., Sharpe A.H. (2010). The PD-1 pathway in tolerance and autoimmunity. Immunol. Rev..

[51] Filion L.G., Izaguirre C.A., Garber G.E., Huebsh L., Aye M.T. (1990). Detection of surface and cytoplasmic CD4 on blood monocytes from normal and HIV-1 infected individuals. J. Immunol. Methods.

[52] Jago C.B., Yates J., Câmara N.O., Lechler R.I., Lombardi G. (2004). Differential expression of CTLA-4 among T cell subsets. Clin. Exp. Immunol..

[53] Li H., Boulougoura A., Endo Y., Tsokos G.C. (2022). Abnormalities of T cells in systemic lupus erythematosus: New insights in pathogenesis and therapeutic strategies. J. Autoimmun..

[54] Gao Z., Feng Y., Xu J., Liang J. (2022). T-cell exhaustion in immune-mediated inflammatory diseases: New implications for immunotherapy. Front. Immunol..

[55] Miggelbrink A.M., Jackson J.D., Lorrey S.J., Srinivasan E.S., Waibl-Polania J., Wilkinson D.S., Fecci P.E. (2021). CD4 T-Cell Exhaustion: Does It Exist and What Are Its Roles in Cancer?. Clin. Cancer Res. Off. J. Am. Assoc. Cancer Res..

